# Evaluating Rosewood Mother & Baby Unit’s Compliance With Vitamin D Monitoring and Prescribing for Mothers

**DOI:** 10.1192/bjo.2025.10547

**Published:** 2025-06-20

**Authors:** Maheen Altaf, Chidi Nwosu, Sujitha Pillay, David Ekone

**Affiliations:** 1 Kent and Medway NHS and Social Care Partnership Trust (KMPT), Dartford, United Kingdom; 2 dartford And Gravesham NHS Trust, Dartford, United Kingdom

## Abstract

**Aims:** To audit the current practices of vitamin D screening and replacement in patients admitted to Rosewood Mother & Baby unit in Kent.

**Methods:** Retrospective audit was conducted to evaluate current practices in screening and treating vitamin D deficiency among patients admitted to mother and baby unit. Relevant standards were set and data collected for patients admitted Jan 2023–Dec 2023. Results presented to stakeholders and improvement plan implemented by circulating trust vitamin D guidelines and discussion with unit dietician. Re-audit was carried out reviewing details of patients admitted from Jan 2024 to Dec 2024.

**Results:** Audit 2023: Out of 60 patients admitted, 95% patients (57) were screened for vitamin D status. 65% of screened patients were either insufficient or deficient (12 insufficient and 27 deficient). Patients received vitamin D replacement as per KMPT guidelines. Aftercare instructions to GP communicated for 93% and 100% patients with vitamin D insufficiency and deficiency respectively. Regarding infant feeding mode 50% were breastfed, 35% on formula milk and 11.6% received mixed feeding. One patient was pregnant and data of one patient not recorded.

Re-audit 2024: Of 56 patients admitted in 2024, 2 patients were not screened. Among 54 patients, 21 and 9 were insufficient and deficient respectively. Out of these 30 patients, 24 were given appropriate supplementation in line with current KMPT clinical guidelines. Aftercare instructions to GP communicated for 17 patients. There was a drop in GP communication and this has been noted for improvement. Four infants were breastfed, 29 on formula milk and 16 received mixed feeding. Three patients were pregnant and for 2 patients the data not recorded.

It is important to highlight that lactating mothers can be deficient in vitamin D. Therefore, this was especially analysed in both audit reports. In both audits, 57 out of 116 patients either breastfed or mix fed the babies. Among these 57 patients, 8 were deficient while 28 were insufficient. This indicates significant (63%) number of patient in both were deficient/insufficient. As vitamin D is implicated in mood and immune system regulation it could play a vital role in the mental state of post-partum patients.

**Conclusion:** This audit/re-audit highlighted need for routine vitamin D screening in patients admitted to psychiatric units especially breastfeeding mothers. It is also important to note that vitamin D could play a vital role in mood and immune system regulation.

